# Biosensing bacterial 16S rDNA by microchip electrophoresis combined with a CRISPR system based on real-time crRNA/Cas12a formation

**DOI:** 10.1039/d2ra03069a

**Published:** 2022-08-10

**Authors:** Feifei Luo, Xing Geng, Zhi Li, Ge Dai, Zhaohui Chu, Pingang He, Fan Zhang, Qingjiang Wang

**Affiliations:** School of Chemistry and Molecular Engineering, East China Normal University 500 Dongchuan Road Shanghai 200241 P. R. China qjwang@chem.ecnu.edu.cn +86 21 54340015

## Abstract

The accurate, simple and sensitive detection of bacterial infections at the early stage is highly valuable in preventing the spread of disease. Recently, CRISPR–Cas12a enzyme-derived nucleic acid detection methods have emerged along with the discovery of the indiscriminate single-stranded DNA (ssDNA) cleavage activity of Cas12a. These nucleic acid detection methods are made effective and sensitive by combining them with isothermal amplification technologies. However, most of the proposed CRISPR–Cas12a strategies involve Cas–crRNA complexes in the preassembled mode, which result in inevitable nonspecific background signals. Besides, the signal ssDNA used in these strategies needs tedious pre-labeling of the signal molecules. Herein, a post-assembly CRISPR–Cas12a method has been proposed based on target-induced transcription amplification and real-time crRNA generation for bacterial 16S rDNA biosensing. This strategy is label-free through the combination of microchip electrophoresis (MCE) detection. In addition, this method eliminates the need for a protospacer adjacent motif (PAM) on the target sequences, and has the potential to be an effective and simple method for nucleic acid detection and infectious disease diagnosis.

## Abstract 2

Real-time crRNA/Cas12a formation derived CRISPR system for bacterial sensing.

## Graphical abstract

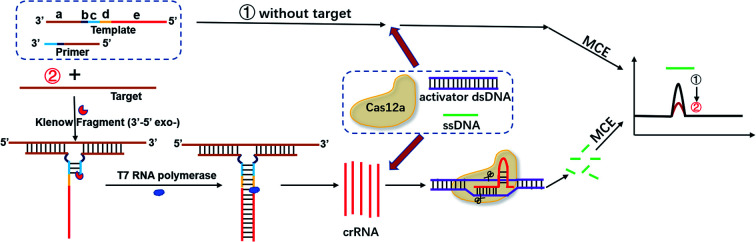

## 1. Introduction

The spread of pathogens in food, water and medical waste is a huge threat to human and animal health, and contributes to approximately one-third of global mortality each year.^1,2^ Despite the discovery of a variety of antibiotics to treat infectious diseases, a delayed diagnosis may lead to the abuse of antibiotics, and in turn increase bacterial resistance to antibiotics.^3–5^ The exploration of accurate, fast and simple methods for pathogenic bacterial detection is crucial in preventing the outbreak of infectious diseases worldwide.^6,7^ Along with the development of molecular diagnostic techniques, the use of nucleic acids as biomarkers for the highly sensitive identification of bacteria has proven to be an effective approach, and many detection methods that target bacterial nucleic acid have been developed.^8–10^

Clustered regularly interspaced short palindromic repeats, together with the associated Cas protein (CRISPR–Cas), are adaptive immune systems in bacteria and archaea for host defense against viral infection.^11–13^ Recently, the CRISPR–Cas system has emerged as a brand-new field to develop novel biosensing platforms for nucleic acid detection since the discovery of its collateral cleavage activity.^14–16^ Cas12a is a type V CRISPR effector protein that can be activated through RNA-guided DNA binding and unleash indiscriminate single-stranded DNA (ssDNA) cleavage activity (trans-cleavage activity) that completely degrades ssDNA molecules.^17,18^ Some nucleic acid detection methods based on the trans-cleavage activity of Cas12a, with high specificity and sensitivity, have been proposed in recent years. For example, a CRISPR–Cas12a-based electrochemical biosensor for HPV-16 detection was developed by Dai *et al.*,^19^ and an experimental limit of detection (LOD) of 50 pM was obtained. Chen *et al.*^20^ proposed a methodology based on the CRISPR–Cas12a system that combined with enhanced strand displacement amplification for human immunodeficiency virus detection achieved an actual sensitivity of sequence-specific DNA of up to 100 aM. Wang *et al.*^21^ developed a one-pot toolbox based on Cas12a/crRNA for *E. coli* O157:H7 and *Staphylococcus aureus* detection; the definite LOD values for *E. coli* O157:H7 and *Staphylococcus aureus* were 1.291 aM and 1.287 aM, respectively.

However, most of the proposed CRISPR–Cas12a sensing methods involve Cas/crRNA complexes in the preassembled mode, which can induce a highly undesired background signal due to their nonspecific trans-cleavage activity even in the absence of target activators.^22^ In addition, tedious pre-labeling of the signal ssDNA is usually needed in these methods to produce the corresponding electrical or fluorescent signals. Microchip electrophoresis LED-induced fluorescence (MCE-LIF) detection can realize efficient nucleic acid separation and has on-line fluorescent labeling capacity, which has been proven to be a powerful tool for nucleic acid analysis.^23–25^ Our group has developed several sensitive bacterial nucleic acid detection methods that combine nucleic acid amplification with MCE-LIF detection.^26–28^

Here, we make full use of the advantages of MCE-LIF detection and the CRISPR–Cas12a system, and propose a transcription amplification-derived real-time crRNA generation CRISPR–Cas12a method (TA-CRISPR–Cas12a) combined with MCE-LIF detection for bacterial 16S rDNA biosensing. In the presence of target DNA, it can specifically hybridize with the template and primer to produce numerous crRNA under the effect of Klenow fragment (3′–5′ exo-) (KF) and T7 RNA polymerase (T7). Then, Cas12a can bind with crRNA and get activated by the designed activator dsDNA to realize the cleavage of the signal ssDNA. As a result, the fluorescence signal of ssDNA decreases in the MCE-LIF detection, so the target DNA can be quantified according to the decrease in the ssDNA signal. This TA-CRISPR–Cas12a-based MCE-LIF strategy is label-free and possesses a post-assembly mode of the CRISPR–Cas12a system, which is simple and effective in bacterial 16S rDNA detection. The detection of *Vibrio parahaemolyticus* is important since it can contaminate seafood and threaten people's health. Here, *Vibrio parahaemolyticus* was used as a model bacterium to verify the principle of the TA-CRISPR–Cas12a-based MCE-LIF strategy. This proposed strategy achieved a LOD of 45 pM in *Vibrio parahaemolyticus* 16S rDNA detection and has the potential for practical infectious diseases diagnosis.

## 2. Experimental section

### 2.1. Materials and instruments

All of the oligonucleotides applied in this study were synthesized and HPLC-purified by Sangon Biotech Co., Ltd. (Shanghai, China), and their detailed sequence information is shown in Table 1. Klenow fragment (3′–5′ exo-) (5000 U mL^−1^) with 10× NEBuffer 2 (100 mM MgCl_2_, 100 mM Tris–HCl, 500 mM NaCl, 10 mM DTT, pH 7.9 @ 25 °C), T7 RNA polymerase (50 000 U mL^−1^) with 10× RNAPol reaction buffer (600 mM MgCl_2_, 400 mM Tris–HCl, 20 mM spermidine, 10 mM DTT, pH 7.9), EnGen Lba Cas12a (Cpf1) with 10× NEBuffer 2.1 (500 mM NaCl, 100 mM Tris–HCl, 100 mM MgCl_2_, 1000 μg mL^−1^ BSA, pH 7.9 @ 25 °C) and DNaseI (RNase-free) with 10× DNaseI reaction buffer (1 mM CaCl_2_, 100 mM Tris–HCl, 25 mM MgCl_2_, pH 7.5 @ 25 °C) were purchased from New England Biolabs (Beijing, China). Recombinant RNase inhibitor was purchased from Takara (Beijing, China). NTP mixture, dNTP mixture, EDTA buffer (0.5 M), Rapid Bacterial Genomic DNA Isolation Kit, DEPC-treated water, 2× SG Fast qPCR Master Mix and 1× TE buffer were obtained from Sangon Biotech Co., Ltd. *Vibrio parahaemolyticus* (ATCC17802) was purchased from Luwei Microbial Sci. & Tech. Co., Ltd. (Shanghai, China). Brain heart infusion (BHI) broth was supplied by Hope Bio-Technology Co. Ltd. (Qingdao, China). SYBR gold nucleic acid gel stain was purchased from Thermo Fisher Scientific. DNA-500kit was supplied by Genesci Medical Technology Co., Ltd. (Shanghai, China). Ultrapure water (18 MΩ cm^−1^ resistivity) was produced by a Milli-Q water purification system and autoclaved (121 °C, 20 min) thoroughly before use.

**Table 1:** The detailed sequences of DNA used in the experiments^a^

Name	Sequence (5′–3′)	Length (nt)
VP-16S	AGGCCACAACCTCCAAGTAGACATCGTTTACGGCGT GGACTACCAGGGTATCTAATCCTGTTTGCTCC	68
Template	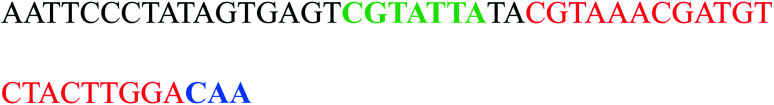	88
Primer	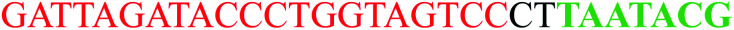	30
dsDNA1	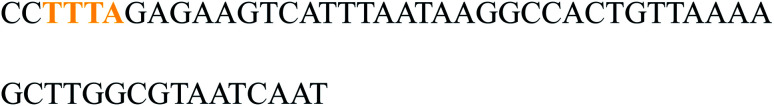	53
dsDNA2	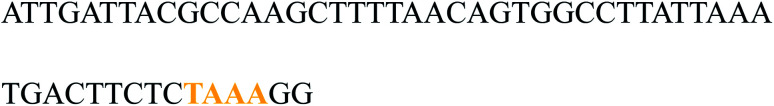	53
ssDNA	ATTCAGTCACGCACG	15
EF-16S	GAAGAACAAGGACGTTAGTAACTGAACGTCCCCTGACGGTATCTAACCAGAAAGCCACGGCTAACTAC	68
SA-16S	GGGAAGAACATATGTTAAGTAACTGTGCACATCTTGACGGTACCTAATCAGAAAGCCACGGCTAACTA	68
EC-16S	GGAGGAAGGGAGTAAAGTTAATACCTTTGCTCATTGACGTTACCCGCAGAAGAAGCACCGGCTAACTC	68
PA-16S	GGAGGAAGGGCAGTAAGTTAATACCTTGCTGTTTTGACGTTACCAACAGAATAAGCACCGGCTAACTT	68
PM-16S	GGAGGAAGGTGATAAGGTTAATACCCTTATCAATTGACGTTACCCGCAGAAGAAGCACCGGCTAACTC	68

a The abbreviation of artificial DNA: VP, *Vibrio parahaemolyticus*; EF, *Enterococcus faecalis*; SA, *Staphylococcus aureus*; EC, *Escherichia coli*; PA, *Pseudomonas aeruginosa*; PM, *Proteus mirabilis*; 16S, 16S rDNA. The green bases are complementary regions between the template and primer. The red bases are complementary regions to VP-16S. The orange bases are the PAM region. The blue bases are non-complementary regions to VP-16S (the sealing bases).

An MCE-202 MultiNA and quartz microchip were supplied by Shimadzu (Kyoto, Japan). A metal bath and constant temperature incubator shaker were purchased from Shanghai Yiheng Scientific Instrument Co., LTD (Shanghai, China). A Bio-Rad CFX96™ Real-Time system was purchased from Bio-Rad Laboratories (Shanghai, China).

### 2.2. Target DNA detection procedures

The target DNA detection procedure consists of two parts, namely the specific generation of crRNA (part A) and the activation of Cas12a to digest the signal ssDNA (part B). The detailed reaction procedure of part A is as follows. Firstly, target DNA with different concentrations, template (100 nM), primer (100 nM), 1× NEBuffer 2, 1× RNAPol reaction buffer, and 3 μL DEPC-treated water were mixed and incubated at 95 °C for 5 min. Then, the mixture was slowly cooled to room temperature to obtain defective T junction hybrids of the target DNA, template and primer. After that, recombinant RNase inhibitor, NTP mixture (0.8 mM), dNTP mixture (0.4 mM), 0.7 μL KF and 0.6 μL T7 were added to the mixture and the mixed solution was incubated at 37 °C for 2.5 h to generate a great amount of crRNA. Subsequently, the remaining DNA was degraded with DNaseI and the reaction was terminated at 80 °C for 10 min. For part B, dsDNA1, dsDNA2, ssDNA, 1× NEBuffer 2.1 and 2.3 μL DEPC-treated water were mixed, followed by incubation at 95 °C for 5 min, and slowly cooled to room temperature to obtain activator dsDNA and signal ssDNA. Then, Cas12a and 3 μL part A solution were added to the part B solution and incubated at 37 °C for 30 min to realize the effective digestion of ssDNA by Cas12a/crRNA. Finally, the part B reaction was terminated at 95 °C for 5 min and detected by MCE-LIF with five times dilution. The detection process of MCE-LIF is the same as that described in our previous study.^27^ All the subsequent experiments were carried out using the standard procedure described here unless otherwise specified.

### 2.3. Bacterial culture and quantification

In this study, sterile BHI broth containing five percent sodium chloride (BHI broth) was used for *Vibrio parahaemolyticus* culture and BHI agar containing five percent sodium chloride (BHI agar) was used for plate-counting. Firstly, *Vibrio parahaemolyticus* was cultured in the BHI broth and placed in an incubator-shaker at 120 rpm overnight at 37 °C. Then, 1 mL of the bacterial suspension was sucked out and centrifuged (8000 rpm, 5 min) to obtain bacterial cells. These bacterial cells were resuspended in 1 mL sterile ddH_2_O after 3 rounds of washing. The viable cell count of each bacterium was determined by plating 10 μL of the appropriate dilution onto BHI agar and counting the corresponding colony forming units (CFUs) after incubation overnight at 37 °C.

### 2.4. 16S rDNA extraction and quantification

According to the manufacturer's instructions for the Rapid Bacterial Genomic DNA Isolation Kit, 1 mL of the cultured *Vibrio parahaemolyticus* suspension was used to extract bacterial 16S rDNA. After a series of procedures, the extracted DNA was resuspended in 50 μL 1× TE buffer and stored at −20 °C for further use. The extracted DNA was quantified by qPCR, and the detailed procedures were consistent with our previous study.^27^

## 3. Results and discussion

### 3.1. Principle of this method

**Fig. 1:**
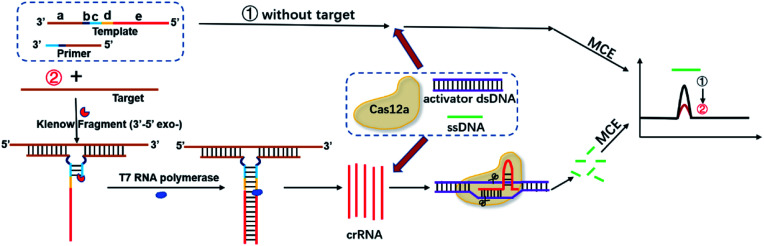
Schematic illustration of the TA-CRISPR–Cas12a and MCE-LIF-based strategy for DNA detection.

Our strategy for bacterial DNA analysis is illustrated in Fig. 1. In this study, a template is suitably designed with five regions: region a, the complementary sequence of target DNA (brown); region b, the site to generate an artificial vacuole (black); region c, the complementary sequence between template and primer (blue); region d, the domain that could form T7 promoter after amplification (yellow); and region e, the complementary sequence of crRNA (red).

In the absence of target DNA, the template and primer barely combine with each other since the complementary sequence between them is very short. Thus, there will be no further amplification, transcription, activation of Cas12a and cleavage of ssDNA, and the signal of ssDNA in MCE-LIF detection will not change (route 1).

When the target DNA is present, it can specifically combine with the template and primer at region a to form a defective T junction structure, and the polymerization will start along the primer under the effect of KF DNA polymerase; as a result, double-stranded DNA that contains the T7 promoter region will form. Then, T7 RNA polymerase will be activated and proceed with the transcription reaction to generate numerous crRNAs. After that, numerous Cas12a/crRNA complexes will be generated, which can recognize the corresponding activator dsDNA to activate the trans-cleavage activity of Cas12a. Finally, the ssDNA with a certain concentration will be efficiently cleaved and the MCE-LIF signal of the ssDNA will decrease (route 2), and the signal change of ssDNA in MCE-LIF detection can be applied for the quantification of target DNA.

### 3.2. Feasibility analysis

Some pre-experiments were conducted to examine the feasibility of our TA-CRISPR–Cas12a strategy for bacterial 16S rDNA detection. Here, synthetic *Vibrio parahaemolyticus* 16S rDNA (VP-16S) was used as a target. The detection procedure consists of two crucial steps, namely the target-induced specific generation of crRNA and the formation of the Cas12a/crRNA/dsDNA complex for the cleavage of ssDNA. Gel electrophoresis and MCE-LIF were used to verify these two crucial steps.

**Fig. 2:**
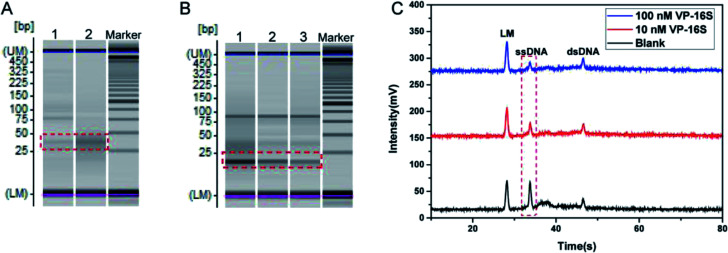
Evaluating the feasibility of the TA-CRISPR–Cas12a strategy for DNA detection. (A) Gel electrophoresis image obtained with different part A reactions. Lane 1, template + primer + recombinant RNase inhibitors + dNTP + NTP + KF + T7 + DNaseI (blank); lane 2, VP-16S (100 nM) + template + primer + recombinant RNase inhibitors + dNTP + NTP + KF + T7 + DNaseI. (B) Gel electrophoresis image obtained with different part B reactions. Lane 1, part A (blank) + Cas12a + ssDNA + dsDNA; lane 2, part A (with 10 nM VP-16S) + Cas12a + ssDNA + dsDNA; lane 3, part A (with 100 nM VP-16S) + Cas12a + ssDNA + dsDNA. (C) Electrophoretogram of the MCE-LIF detection results of different part B solutions. LM: lower markers.

First, the part A reaction was investigated. As shown in Fig. 2A, when the target DNA VP-16S was not added to the part A reaction system, there was only a faint crRNA band in the gel electrophoresis image (lane 1). This is in accordance with the principle that the template and primer barely hybridize in the absence of VP-16S. When the target DNA VP-16S was added to the part A reaction system, a distinct crRNA band appeared (lane 2), indicating the specific formation of crRNA in the presence of VP-16S.

Then, the activation of Cas12a and the subsequent cleavage of ssDNA were evaluated. As displayed in Fig. 2B, when the reaction of part A that did not contain VP-16S finished, and the final part A mixture was added to the part B reaction system, there was a very bright band of ssDNA in the gel electrophoresis image of the final part B mixture (lane 1). However, when the part A system that contained 10 nM VP-16S finished its reaction, and the final mixture was added to the part B reaction system, a faded ssDNA band was observed in the final part B mixture analysis (land 2). When 100 nM VP-16S was added to the part A system to induce a reaction, and the reaction mixture was then added to the part B system, there was a more faded ssDNA band in the gel electrophoresis image (lane 3). All these experimental results demonstrate the successful activation of Cas12a and the cleavage of ssDNA. Besides, MCE-LIF was applied to detect the corresponding reaction mixture of part B, and the peak intensity of ssDNA decreased with the increase in VP-16S (Fig. 2C). Taken together, these experimental results demonstrate that the TA-CRISPR–Cas12a strategy is versatile for nucleic acid detection.

### 3.3. Optimization of the conditions

**Fig. 3:**
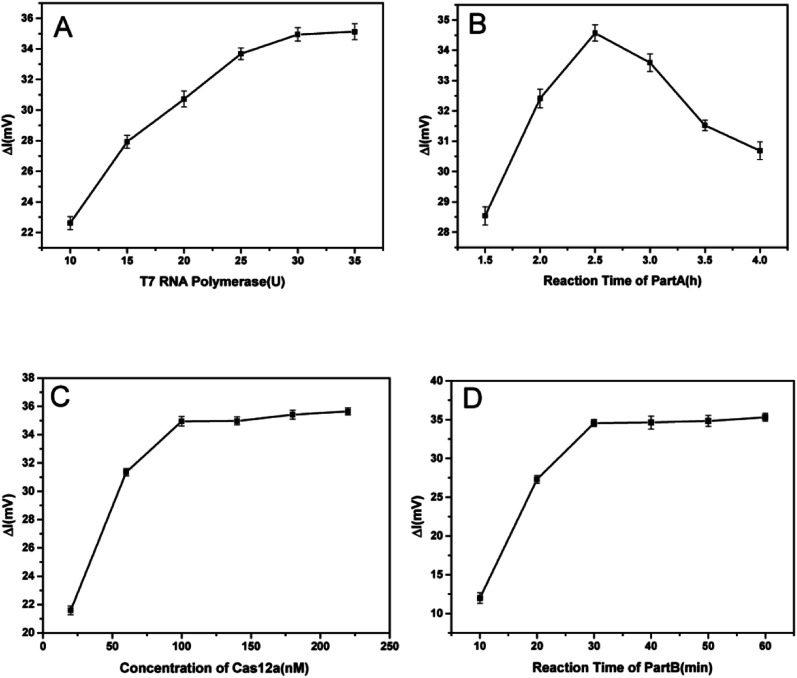
Optimization of the TA-CRISPR–Cas12a experimental conditions, including the (A) quantity of T7 RNA polymerase; (B) reaction time of part A; (C) concentration of Cas12a; and (D) reaction time of part B (cleavage time of Cas12a on ssDNA). Error bars represent SD (*n* = 3).

The amounts of T7 RNA polymerase and Cas12a, the reaction time of part A and the reaction time of part B (cleavage time of Cas12a on ssDNA) were optimized to obtain the best analytical performance of the TA-CRISPR–Cas12a-based MCE biosensing strategy. Here, the amount of Klenow fragment (3′–5′ exo-) was 3.5 U and the concentration ratio of the template and primer was 1 : 1, as per our former experiments.^27^ All optimal experiments were conducted with 100 nM target VP-16S in comparison with a blank control. As shown in Fig. 3A, the Δ*I* (the change in fluorescence intensity, net fluorescence intensity of ssDNA) increased gradually with the increase in T7 RNA polymerase from 10 U to 30 U. When the amount of T7 RNA polymerase crossed 30 U, the Δ*I* did not increase significantly, and 30 U T7 RNA polymerase was chosen for the subsequent experiments. Then, six reaction times of part A, *i.e.* 1.5 h, 2 h, 2.5 h, 3 h, 3.5 h and 4 h, were optimized. As displayed in Fig. 3B, the maximum Δ*I* was obtained at the 2.5 h reaction time of part A. The reason is that with the increase in part A reaction time, the cleavage of ssDNA was more. However, when the part A reaction crossed 2.5 h, the background signal increased significantly, resulting in the decrease in Δ*I*. Hence, 2.5 h was selected as the part A reaction time. As depicted in Fig. 3C, with the increase in Cas12a, Δ*I* increased rapidly and reached a plateau beyond 100 nM. Therefore, 100 nM of Cas12a was selected as the optimal concentration for subsequent experiments. Finally, different reaction times of part B (10 min, 20 min, 30 min, 40 min, 50 min and 60 min) were tested to obtain the optimal ssDNA cleavage time. As shown in Fig. 3D, the Δ*I* increased gradually when the part B reaction time increased from 10 min to 30 min, and reached a plateau beyond 30 min. Therefore, 30 min was chosen as the optimal part B reaction time for the subsequent studies.

### 3.4. Detection performance of the TA-CRISPR–Cas12a strategy

**Fig. 4:**
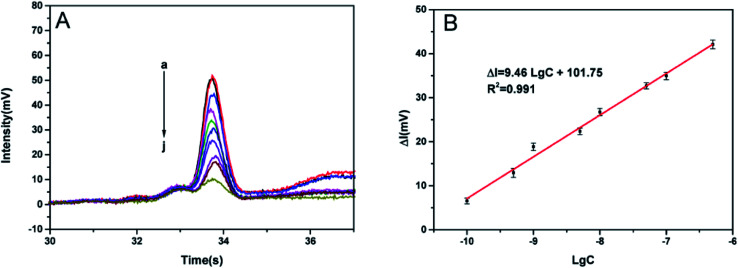
(A) Fluorescence intensity of ssDNA with different concentrations of VP-16S in the reaction system; (a) blank; (b) 1.0 × 10^−11^ M; (c) 1.0 × 10^−10^ M; (d) 5.0 × 10^−10^ M; (e) 1.0 × 10^−9^ M; (f) 5.0 × 10^−9^ M; (g) 1.0 × 10^−8^ M; (h) 5.0 × 10^−8^ M; (i) 1.0 × 10^−7^ M; (j) 5.0 × 10^−7^ M. (B) Linear relationship between the net fluorescence intensity of ssDNA and the logarithm of VP-16S concentration. Error bars represent SD (*n* = 3).

Under the optimal experimental conditions, target VP-16S at various concentrations was detected by the TA-CRISPR–Cas12a-based MCE-LIF detection strategy. As shown in Fig. 4A, a gradual decrease in the ssDNA fluorescence intensity was observed as the VP-16S concentration increased from 1.0 × 10^−11^ M to 5.0 × 10^−7^ M. The Δ*I* was linearly related to the logarithm of VP-16S concentration ranging from 1.0 × 10^−10^ M to 5.0 × 10^−7^ M (Fig. 4B), with a regression equation of Δ*I* = 9.46 lg *C* + 101.75 (*R*^2^ = 0.991), in which *C* is the concentration of VP-16S. The limit of detection (LOD) was calculated to be 45 pM according to a traditional and standard approach,^29^ indicating the satisfactory sensitivity of this TA-CRISPR–Cas12a-based MCE-LIF detection strategy. As displayed in Table 2, our proposed new strategy had better or comparable sensitivity to some reported DNA detection methods.

**Table 2:** Comparison of other DNA detection methods with our TA-CRISPR–Cas12a-based MCE-LIF strategy

Methods	LOD	References
CRISPR–Cas12a-based electrochemical biosensor	50 pM	19
Fluorescent biosensor based on DNA self-assembly-activated hemin-mimetic enzyme	78 pM	30
Fluorescence measurements based on palindrome-mediated strand displacement amplification	50 pM	31
CRISPR–Cas system-enhanced electrochemical DNA sensor	31.9 pM	32
Fluorescence measurements based on reverse strand displacement amplification	1 nM	33
TA-CRISPR/Cas12a-based MCE-LIF strategy	45 pM	This work

### 3.5. Selectivity of this strategy

**Fig. 5:**
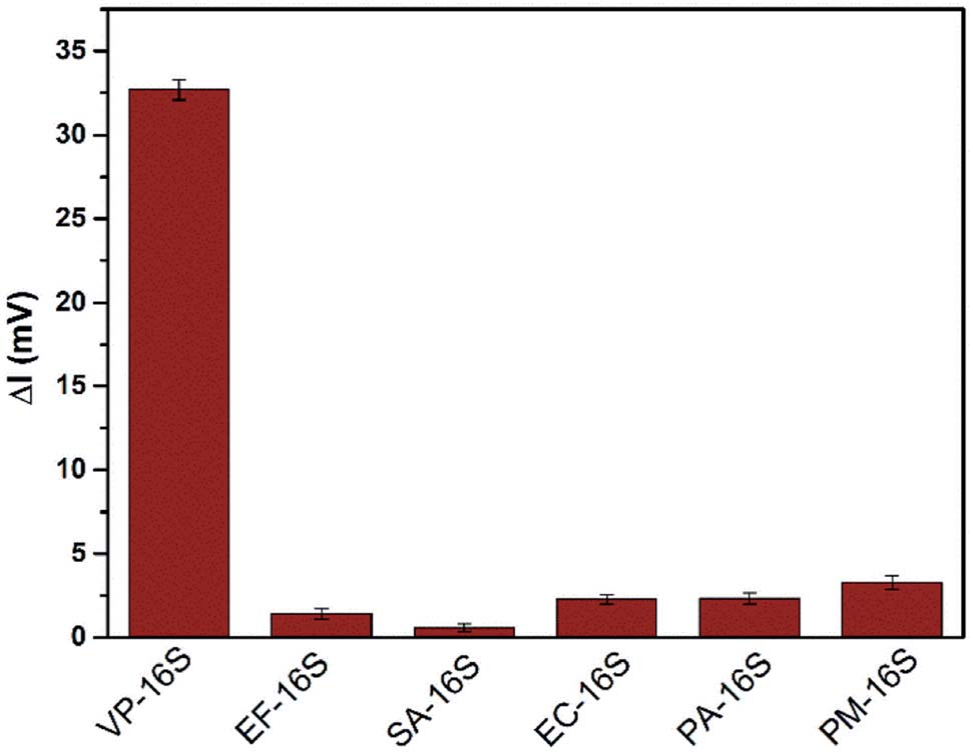
Net fluorescence responses of VP-16S and other bacterial 16S rDNA (all at 100 nM).

Here, five synthetic bacterial 16S rDNAs, namely *Enterococcus faecalis*, *Staphylococcus aureus*, *Escherichia coli*, *Pseudomonas aeruginosa* and *Proteus mirabilis*, were used to evaluate the selectivity of the TA-CRISPR–Cas12a-based MCE-LIF detection strategy for VP-16S analysis. The DNA concentration used in these experiments is 100 nM. As shown in Fig. 5, when other interfering bacterial 16S rDNAs were detected using this TA-CRISPR–Cas12a-based MCE-LIF detection strategy, the net fluorescence signal of the ssDNA is negligible in comparison to that of VP-16S detection. This demonstrates the satisfactory specificity towards the target VP-16S of this newly proposed strategy.

### 3.6. Analytical performance of the method towards real samples

**Fig. 6:**
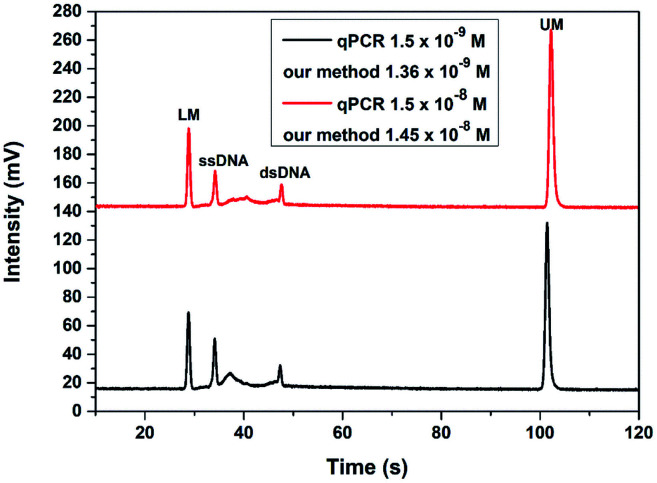
The detection results of two different concentrations of bacterial genomic DNA with the TA-CRISPR/Cas12a-based MCE-LIF strategy. LM: lower markers, UM: upper markers.

To verify the application of this TA-CRISPR–Cas12a-based MCE-LIF detection strategy in bacterial 16S rDNA detection, two genomic DNA samples with different concentrations extracted from *Vibrio parahaemolyticus* were analyzed. As shown in Fig. 6, the detection results with our proposed method were calculated to be 1.36 × 10^−9^ M and 1.45 × 10^−8^ M according to the peak height of ssDNA, which is in agreement with the quantification results of qPCR (1.5 × 10^−9^ M and 1.5 × 10^−8^ M, respectively). The relative standard deviations (RSDs) of three parallel experiments were 5.93% and 4.61%, respectively. These results demonstrate that our proposed strategy is accurate and applicable for the detection of extracted bacterial DNA samples.

## 4. Conclusions

In summary, a bacterial 16S rDNA detection method that combines the transcription amplification-derived real-time crRNA generation CRISPR–Cas12a system with MCE-LIF detection has been proposed. Compared with traditional CRISPR–Cas12a-based assays, our developed TA-CRISPR–Cas12a-based MCE-LIF detection strategy involves real-time crRNA generation and the crRNA/Cas12a post-assembly mode, which could eliminate the highly undesired background signal. Besides, the signal ssDNA applied in the TA-CRISPR–Cas12a-based MCE-LIF detection strategy is label-free, which is cheaper and simpler. This proposed strategy has good selectivity and real genomic sample utility, demonstrating huge potential in infectious diseases diagnosis.

## Conflicts of interest

There are no conflicts to declare.

## Supplementary Material
